# American Rhinological Association

**Published:** 1885-09-20

**Authors:** 


AMERICAN RHINOLOGICAL ASSOCIATION.—This Association will be held at Lexington, Ky., on October 6, 1885. The papers and discussions will be devoted exclusively to diseases of the nasal organs. P. W. Logan, M. D., of Knoxville, is president, and C. A. S. Sims, M. D., of St. Joseph, Mo., is secretary. The vice-presidents are A. DeVilbis, M. D., of Toledo, Ohio, and J. A. Stucky, M. D., of Kentucky.

